# Phenotypic characterisation and production system of the Nordestino horse from a multivariate perspective

**DOI:** 10.1038/s41598-023-51018-y

**Published:** 2024-01-12

**Authors:** Neila Lidiany Ribeiro, Geovergue Rodrigues de Medeiros, Núbia Michelle Vieira da Silva, Kilmer Oliveira Soares, Janaína Kelli Gomes Arandas, George Vieira do Nascimento, Maria Norma Ribeiro

**Affiliations:** 1 Pesquisador bolsista PCI/CNPq do Instituto Nacional do Semiárido - INSA, Campina Grande, Paraíba Brazil; 2 Pesquisador do Instituto Nacional do Semiárido - INSA, Campina Grande, Paraíba Brazil; 3 Universidade Federal Rural de Pernambuco - UFRPE, Recife, Pernambuco Brazil

**Keywords:** Developmental biology, Ecology, Systems biology, Climate sciences

## Abstract

Zootechnical data is a big challenge in the extensive rearing system of Brazilian locally adapted breeds once smallholdings with limited resources and funds rear them. So, information on Brazil's breeding system of locally adapted breeds is still scarce; this situation is more challenging for equine breeds**.** The present study aimed to describe the local rearing systems and the phenotypic profile of the Nordestino horse breed in Paraíba state and contribute to breed conservation. Data from males (entire and castrated) and females from 50 municipalities in Paraíba state were used. Two hundred sixty-nine animals (111 females, 121 castrated males, and 37 entire males) from 129 breeders were analyzed. A questionnaire consisting of direct and objective questions was applied to understand the breeding system adopted. There was a predominance of the extensive breeding system (85%), which reflects the adaptation of the Nordestino Horse to the region's natural conditions. The lower frequency of use of cultivated pastures may be related to issues of economic viability since the maintenance of cultivated pastures may require additional investments compared to the use of natural pastures. Entire males had a minimum withers height (WH) of 135 cm. Of the 11 morphometric measurements, only five were considered discriminating by the stepwise analysis. The remaining Nordestino horses have morphological characteristics within the breed standard.

## Introduction

Several horse breeds were developed and adapted to different Brazilian regions over the centuries, meeting local needs. Among these breeds, the *Nordestino* horse stands out: a small to medium-sized trotting gait, light in general appearance, well-proportioned, small and robust hooves, sound resistance, and adapted to the semiarid region^1^. The semiarid region is characterized by a negative water balance resulting from an average annual precipitation of lower than 800 mm, insolation of 2800 h per year, an average annual temperature ranging from 23 to 28 °C, evaporation of 2000 mm per year, and a relative humidity around 60%^2^.

Most horses in the Northeastern semiarid region are animals without a defined breed pattern, remnants of breeds resulting from crossings with other breeds, which managed to adapt to the bioclimatic conditions of the region^1^. Today, the crosses mentioned above are with the Quarto de Milha, Mangalarga, and English Thoroughbred horse breeds. Indiscriminate crossbreeding, the castration of males, and using females in stud farms as recipients keep the breed under constant threat, so a genetic conservation plan is necessary.

Phenotypic characterization is one of the first steps in conserving animal genetic resources programs since the results obtained from zoometric measurements allow for characterizing or classifying individuals within a population or breed. Body size and body conformation are essential traits to characterize horse breeds. Breeders' associations typically select horses based on functional criteria and encourage breeding animals with the best available body structure, with correct skeletal conformation being a key determinant of body type^3^. According to Parés-Casanova^4^ and Pimentel et al.^5^, linear measurements have been used for selection, breed differentiation, and identification of specific abilities of each breed, as they can contribute to verifying the qualities and defects of each body region. Zootechnical selection should instead be carried out within the breed to correct defects that could harm and prevent its use^6^.

Due to the lack of information, the present study aimed to describe the breeding systems and characterize the phenotypical profile of the Nordestino horse in Paraiba state, seeking to contribute to breed conservation.

## Materials and methods

### Animals and locals of data collection

**Figure 1:**
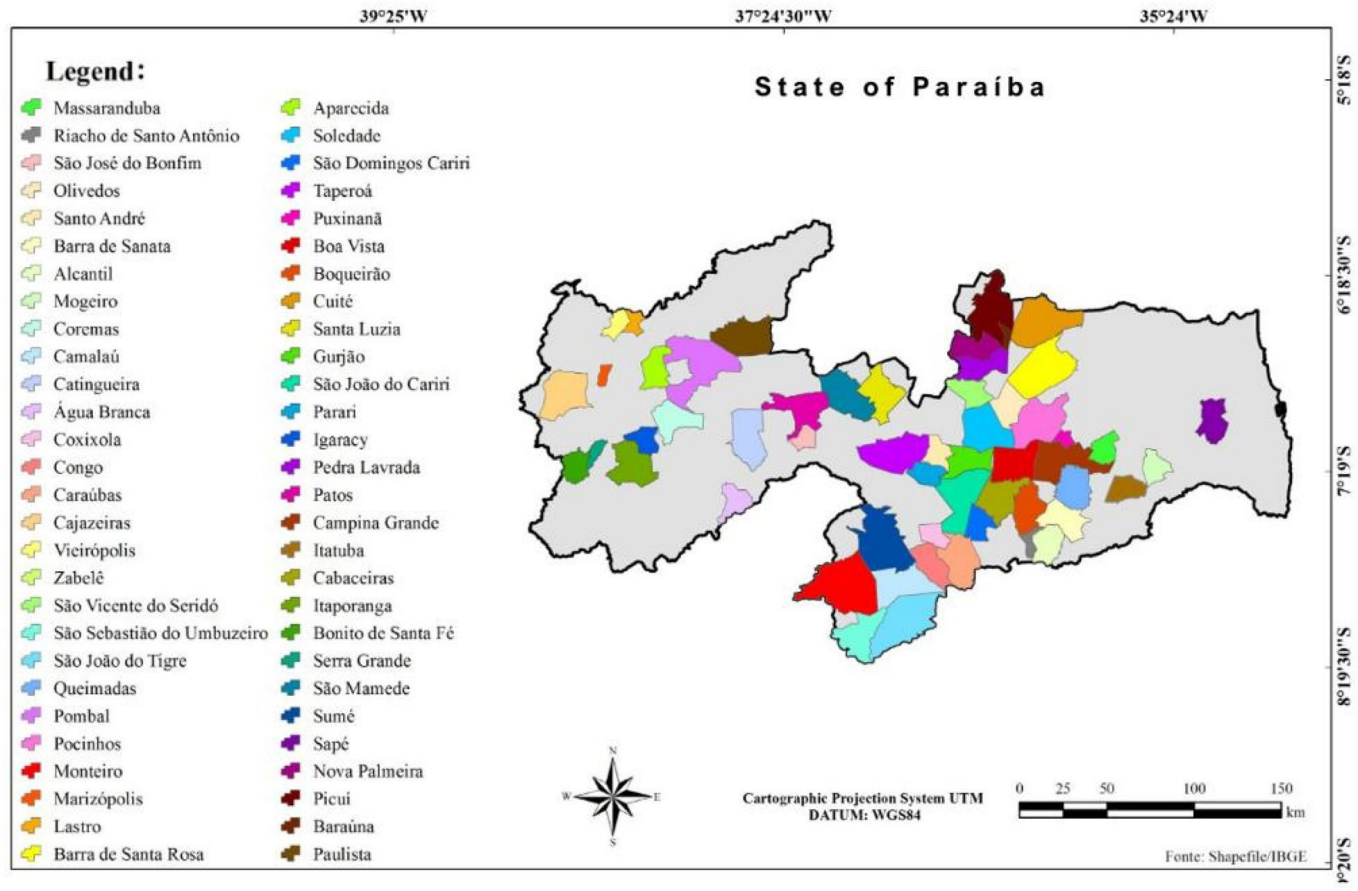
Mapa state of Paraiba/Brazil.

This study was carried out following the ethical principles of animal experimentation and under the approval of the Animal Ethics Committee of the *Instituto Nacional do Semiárido* (National Institute of Semi-Arid) (protocol n° 0002/2022), Brazil. Data from males (entire and castrated) and females from 50 municipalities in Paraíba state were used (Fig. 1).

**Figure 2:**
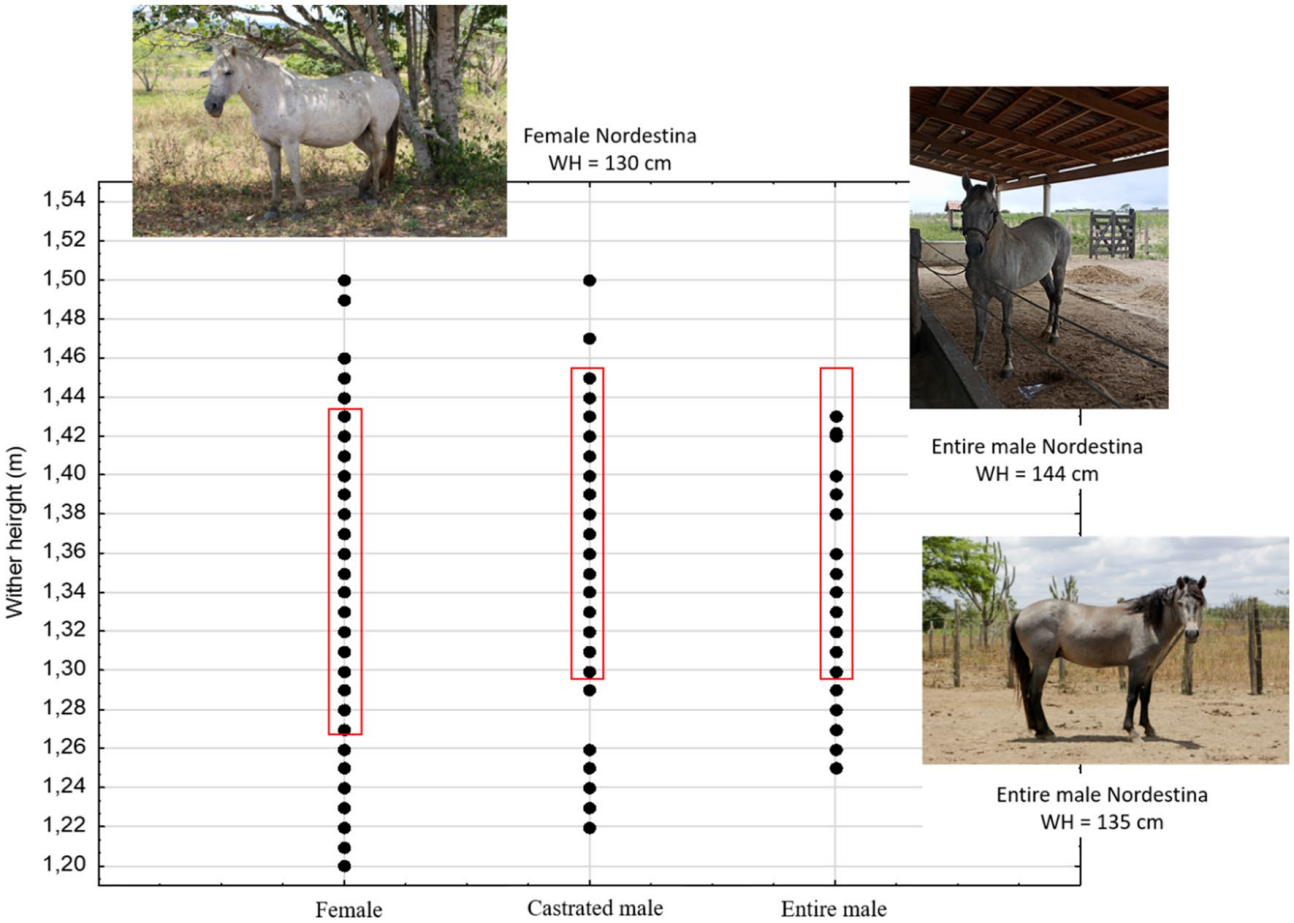
Selection protocol for animals used in the research considering whether height.

The initial data file was composed of information referring to 310 animals. However, after preliminary data analysis, it was decided to use animals whose height was within the breed standard described by the *Associação Brasileira de Criadores do Cavalo Nordestino* (Brazilian Association of Breeders of the *Nordestino* horse) (ABCCN) (Fig. 2). Therefore, data from 269 animals (111 females, 121 castrated males, and 37 entire males) were analyzed. The animals were evaluated by dental chronometry; all were in their first molt and entire mouth. Table 1 shows the number of animals and the wither height (WH) of animals excluded from the study, considering the minimum and maximum WH.

**Table 1:** Withers height (WH) values of animals excluded from the study.

Sex	Number of animals	Minimum and maximum value WH	Average WH
Female	16	1.20–1.26	1.24
	6	1.44–1.50	1.46
Castrated male	5	1.23–1.29	1.25
	3	1.47–1.50	1.48
Entire male	11	1.26–1.29	1.28

### Data collection of the breeding system

Interviews were carried out with 128 breeders using a questionnaire composed of direct questions adapted from Arandas et al.^7,8^. To guarantee uniformity of the data collected, all questions, answer options, and the sequence of questions were identical for all interviewees. To ensure that the differences in the results obtained were due to each breeder's vision, the interviewers were trained adequately through in-person and virtual workshops.

The questionnaire was subdivided into sections, whose questions addressed the profile of the breeders, the general traits of the farms, quantitative characteristics of farm infrastructure, qualitative aspects of the herds—food management, health and reproductive, technological traits of the farms; zootechnical management and control.

### Morphometric traits measurements

It was used a tape measure and a hypometer to measure the variables according to Melo et al.^1^:

The measurements performed were:

Wither height (WH) corresponding to the highest point of the interscapular region to the ground;

Croup height (CH) from the highest point of the sacral tuberosity of the ileum to the ground;

Body length (BL) traced between the greater tubercle of the humerus and the ischial tuberosity;

Neck length (NL) distance between the nape of the neck and the superior junction with the withers;

Substernal void height (SVH) distance between the ground and the sternum;

Thoracic depth (TD) vertical distance between the highest point of the interscapular region and the xiphoid cartilage perpendicularly to the ground;

Croup length (CL) vertical distance between the highest point of the croup and the flank crease perpendicular to the ground;

Cannon girth (CaG) was measured by the circumference of the left metacarpal bone in its middle third;

Thoracic perimeter (TP) circumference of the chest traced along the line of the spinous apophysis of the 7th-8th thoracic vertebra to the corresponding lower sternal region at the level of the olecranon tuberosity;

Head length (HL) corresponds to the distance from the occipital protuberance to the most rostral point of the upper lip;

Head width (HW) was the distance between the free part of the right supraorbital border and the left border.

### Zoometric indices

The following zoometric indices were calculated:

Dactyl-thoracic index (DTI = CaG/TP): the relationship between the cannon girth (CaG) and the thoracic perimeter (TP). It should not be less than 0.105 for light horses, 0.108 for intermediate horses, 0.110 for light traction horses, and 0.115 for heavy traction horses^9^;

Body index (BI = BL/TP × 100): the relationship between body length and thoracic perimeter multiplied by 100. Allows classifying animals, considering the Baronian system, into brevilines when less than or equal to 85, mediolines if between 86 and 88, and long when greater than or equal to 90^4^;

Conformation index (CFI = TP^2^/WH): the ratio between the thoracic perimeter squared and divided by the height at the withers. The value of 2.1125 is ideal for the saddle horse, while higher values indicate animals suitable for traction^9^;

Compactness index (COI = (W/WH/100): the relationship between weight (kg) and height at the withers, in cm. For a heavy draft horse, the value must be at least equal to 3.15; for a horse with light traction, 2.75; and, for the saddle horse, 2.60^9^;

The ratio between height at withers and croup (WCR = WH/CH): the ratio between height at withers divided by height at the croup. The value obtained must be equal to 1.00, which expresses a balance factor^9^;

Load index 1 (LOI1 = (TP^2^ × 56)/WH): thoracic perimeter squared and multiplied by the constant 56, divided by the wither height. It expresses the weight, in kilograms, that the animal can support without excessive effort on its back, working at a trot or gallop^9^;

Load index 2 (LOI2 = (TP^2^ × 95)/WH): thoracic perimeter squared and multiplied by the constant 95, divided by the wither height. It expresses the weight, in kilograms, that the animal can support without excessive effort on its back, working at a pace^9^;

Observed body weight (OBW): body weight obtained with a measuring tape when measuring thoracic perimeter;

Estimated body weight (EBW) (W3 = TP^3^ × 80) corresponds to the thoracic perimeter cubed and multiplied by the constant 80^9^.

### Statistical analysis

Data relating to the characterization of the production system were subjected to a frequency analysis. A simple descriptive analysis was conducted for morphometric data, calculating means and standard deviations. The t-test with a significance level of 5%^10^ was applied.

The morphometric traits were submitted to discriminant analysis using the *stepwise* method. The animals were categorized into three groups: Female, Castrated Male, and Whole Male. Multivariate discriminant analysis was chosen as the main statistical approach to identify significant morphometric variables in group differentiation. The stepwise method, an iterative approach that incorporates or removes variables sequentially based on statistical criteria, was chosen. The final discriminant model, composed of the most relevant variables, was developed through this process.

The general form of the discriminant model is expressed as:

$$Y = a_{0} + a_{1} X_{1} + a_{2} X_{2} + \ldots + a_{p} X_{p}$$

where *Y* was the discriminant trait; $a_{0} ,a_{1} ,a_{2} , \ldots ,a_{p}$ coefficients to be estimated; $X_{1} ,X_{2} , \ldots ,X_{p}$ independent traits (morphometrical traits).

### Arrive

The study is reported by ARRIVE guidelines (https://arriveguidelines.org).

## Results

### Characterization of the hearing system

Most breeders adopt the extensive breeding system (85%), where most animals (80%) consume only native pasture (Table 2), which is characteristic of the local production system.

**Table 2:** Frequency of breeders adopting or not adopting feed, health, and reproductive management practices for the *Nordestino* horse in Paraíba State.

Management practice		Yes (%)	No (%)
*Feeding management*			
Cultivated pasture		20	80
Strategic feeding		23	77
Feed supplementation		25	75
Mineral supplementation		48	52
*Reproductive management*			
Zootechnical Bookkeeping		10	90
Controlled mating		100	0
Stallion replace		58	42
Selection		10	90
Castrated		87	13
Taming		10	90
*Sanitary management*			
Strategic deworming		65	35
Periodic tests and EIA control		10	90
Ectoparasite control		10	90

The predominance of the extensive breeding system reflects the adaptation of the Nordestino horse to the local conditions. This result is consistent with the traditional characteristics of the breed, known for its rusticity and resistance. The lower frequency use of cultivated pastures may be related to issues of economic viability since the maintenance of cultivated pastures may require additional investments compared to the use of natural pastures.

Most breeders (88.27%) have between one and three animals (Table 3) used in carts, cultivation, or cattle management; this requires specific management for these animals, as they require different sanitary and reproductive food management strategies than those applied to cattle. The small number of animals per breeder is typical in systems where the horse was used only for handling and managing livestock, unlike horse breeding for other purposes.

**Table 3:** Number of animals (NA), number of breeders (NB) and percentage of breeders (%B).

NA	NB	%B
1	55	42.96
2	43	33.59
3	15	11.72
4	6	4.69
5	3	2.34
6	2	1.56
7	2	1.56
8	1	0.79
9	1	0.79
269	128	100

**Figure 3:**
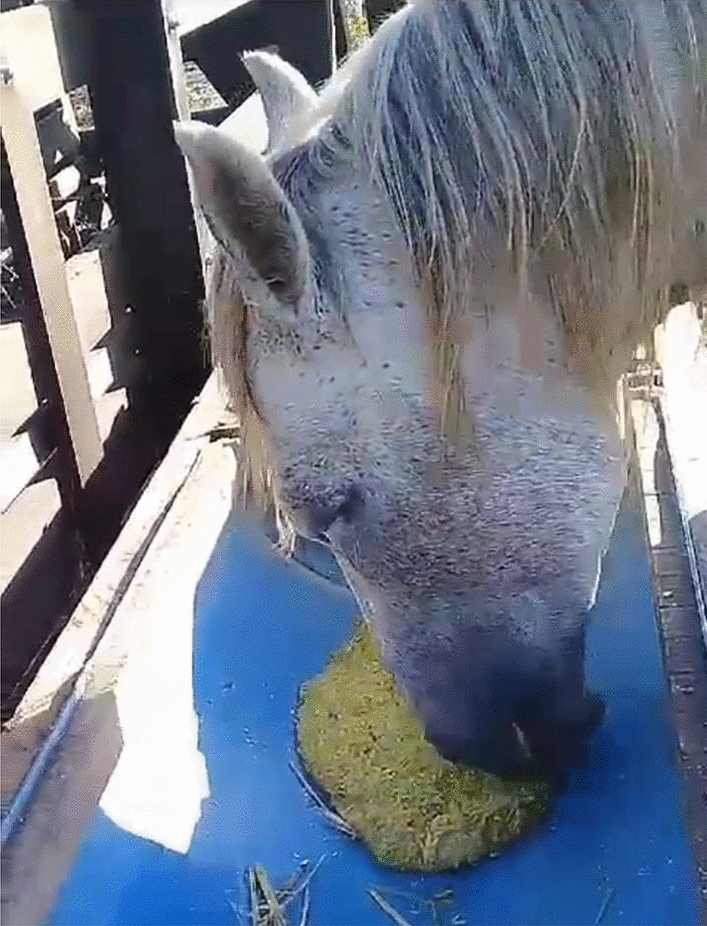
Nordestino horse receiving cactus spear (*Opuntia stricta*) in trough.

Regarding nutritional aspects, horses were kept together with cattle in large areas of native pastures comprising the food base. Twenty-three percent of the interviewed reported the use of mineral or food supplies. Twenty-five percent use concentrate, hay, silage, or corn in supply. A breeder said using a mix of palm Mexican Elephant Ear (*Opuntia stricta*) and corn bran (in the morning shift) (Fig. 3) and bran made from Maniçoba hay (*Manihot pseudoglaziovii*) (in the afternoon shift).

**Figure 4:**
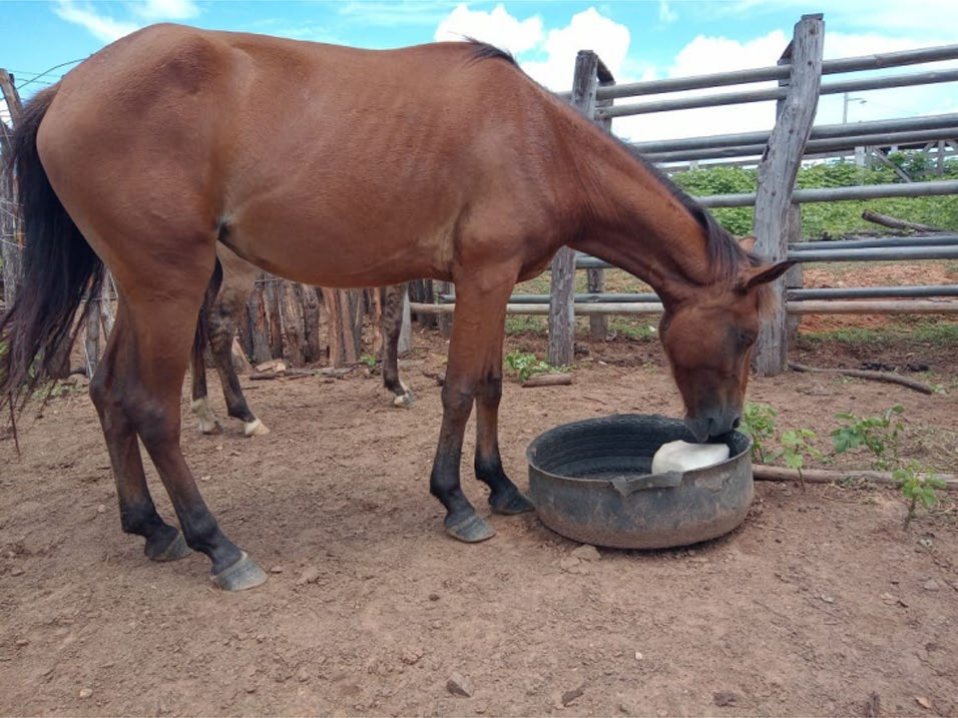
Female receiving mineral supplementation in block form.

**Figure 5:**
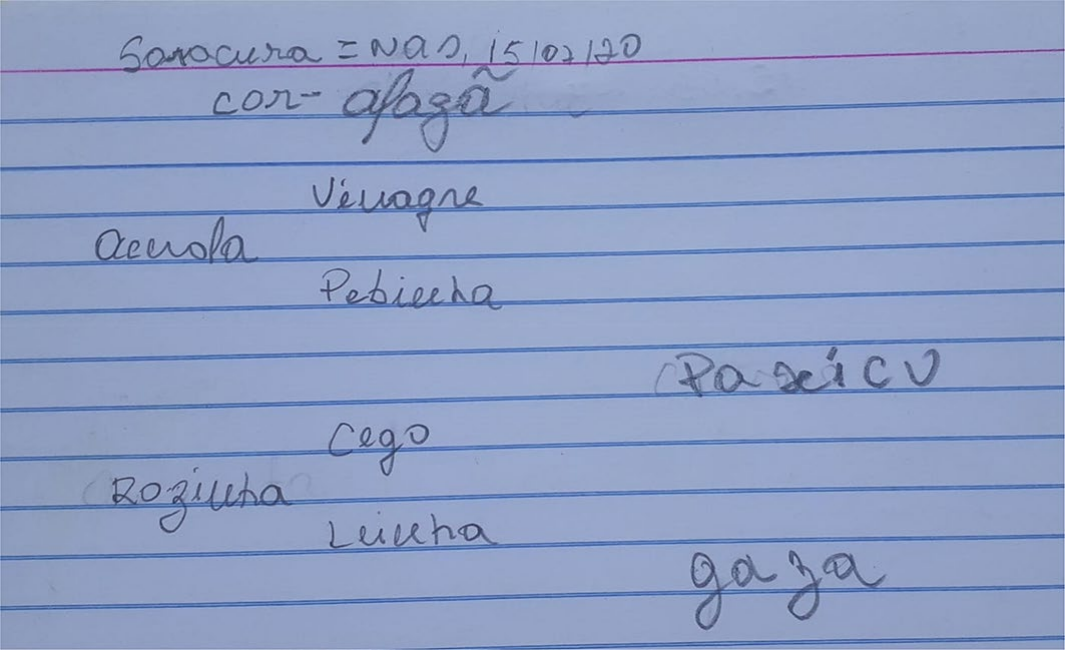
Zootechnical and pedigree control form adopted by one of the breeders interviewed.

Many breeders (48%) stated that they provide mineral supplementation for their animals (Fig. 4). Considering zootechnical records, only 10% of breeders use birth notes and genealogical information (Fig. 5).

**Figure 6:**
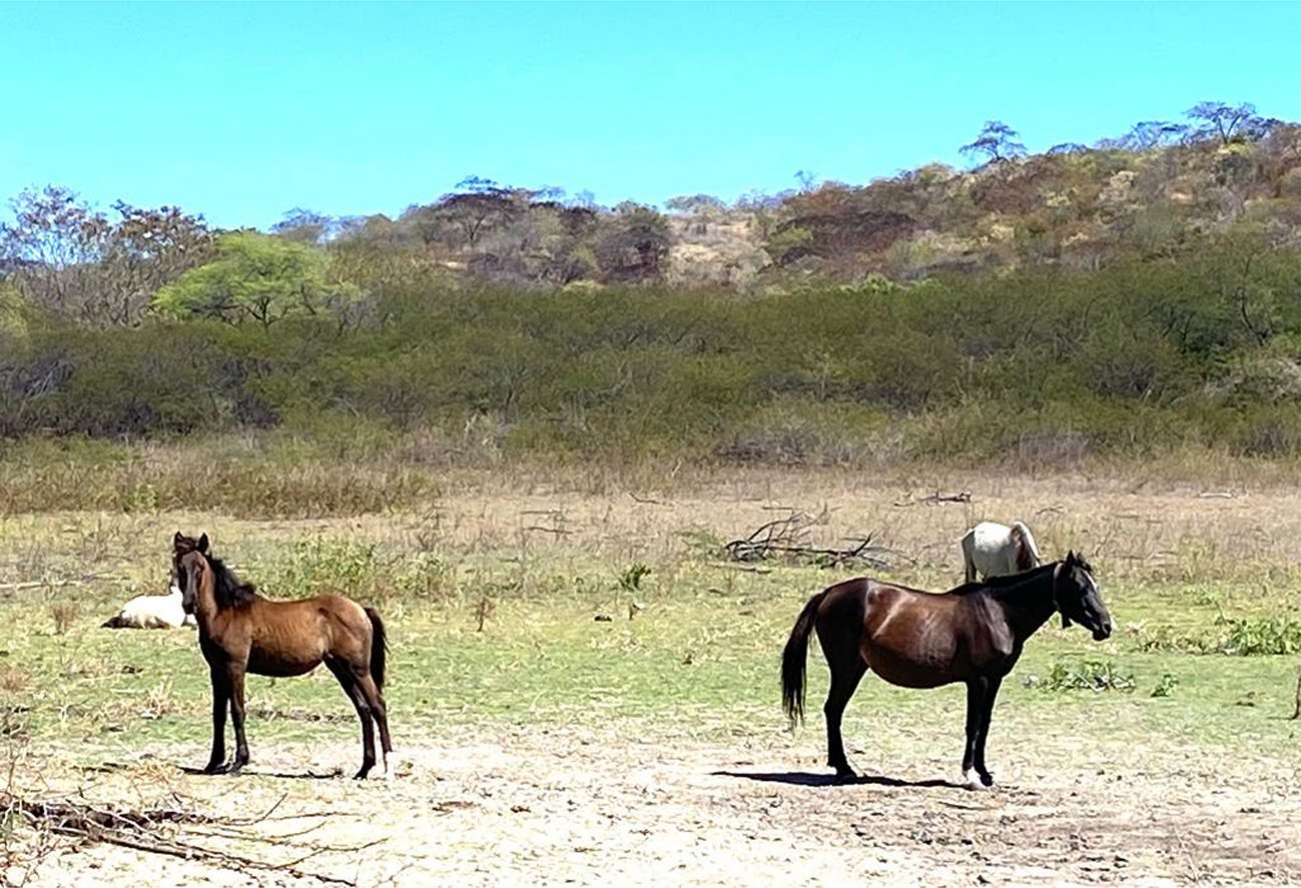
Female *Dalila* is the daughter of *Lobo Mal* stallion with *Cigana* female.

Most breeders adopt controlled breeding as they have few animals, which helps prevent inbreeding. A minority of breeders split animals by sex to avoid endogamic mating and exchanging stallions (58%). This practice was observed on-farm, as seen in Fig. 6. Only 10% of breeders carry out selection. Those who adopt this practice do so based on conformation criteria, such as animal size (from small to medium), suitable aplomb, transmission of the main characteristics to the offspring, age, and number of offspring per breeder.

Rational taming was adopted by only 10% of breeders, whether sending their animals to specialized tamers or a skilled cowboy to carry out the process. On the other hand, it was found that 90% of breeders face difficulties in finding a tamer, resulting in the option for traditional taming or castration.

Regarding sanitary management, it was found that 65% of breeders adopt strategic deworming at the end of the drought and at the beginning of the rainy season when the animals are dewormed. However, only 10% of the breeders said they carried out ectoparasite control and periodic examinations to detect Equine Infectious Anemia (EIA).

### Phenotypic profile of animals

It was observed that neck length (*P* = 0.0044), body length (*P* = 0.0449), thoracic perimeter (*P* = 0.0496), cannon girth (*P* = 0.0029), thoracic depth (*P* < 0.0001), and croup height (*P* = 0.0003) showed significant differences depending on sex (Table 4).

**Table 4:** Mean ± standard deviation of morphometric measurements of the *Nordestino* horse in Paraíba state, according to the animal's sex.

Variables	Female (110)	Castrated male (121)	Entire male (37)	*P*-value
Head length	49.19 ± 3.99	50.03 ± 3.57	49.59 ± 4.43	0.3116
Head width	17.05 ± 1.54	17.16 ± 1.44	16.97 ± 1.74	0.7408
Neck length	60.21 ± 5.08ª	61.22 ± 3.83ª	58.21 ± 6.63b	0.0044
Wither height	1.35 ± 0.04	1.37 ± 0.04	1.35 ± 0.04	0.4360
Croup height	1.35 ± 0.05	1.35 ± 0.05	1.34 ± 0.05	0.4540
Body length	1.36 ± 0.07ª	1.35 ± 0.06ab	1.33 ± 0.06b	0.0449
Thoracic perimeter	1.54 ± 0.10ab	1.57 ± 0.09ª	1.53 ± 0.11b	0.0496
Cannon girth	0.177 ± 0.01b	0.184 ± 0.01ª	0.181 ± 0.01a	0.0029
Substernal void height	65.19 ± 3.54b	69.60 ± 4.80ª	67.70 ± 4.63a	< .0001
Thoracic depth	59.84 ± 8.12ª	61.47 ± 6.76ª	55.78 ± 6.60b	0.0003

Distinct letters in the row differ from each other by the *t*-test at a 5% significant level.

Females and castrated males had greater neck length than all males (*P* = 0.0044). Females had greater body length and were castrated, and all males had statistically equal body length values (*P* = 0.0449). Castrated males had a larger thoracic perimeter than females and all males. Cannon girth (*P* = 0.0024) and height at substernal void (*P* < 0.001) were more significant in castrated males. On the other hand, female and castrated males presented higher values of thoracic depth (*P* = 0.0003) than all males. This results in good lung development and greater digestive capacity, which is of great importance for the performance of the animals. In the case of entire males, the values may be associated with the lower body weight observed in these animals.

In this study, one of the criteria was to evaluate entire males with a minimum withers height (WH) of 130 cm, a value that is within that established by the *Associação Brasileira de Criadores do Cavalo Nordestino* (Brazilian Association of Breeders of the Nordestino Horse)^11^, which is 130–138 cm, with 146 cm being the maximum height allowed by the current regulations of the association as mentioned earlier. Animals with WH below 130 cm are considered minor. However, they are the most commonly found in the different municipalities studied. Genetic factors and management seem responsible for the difficulty in the Nordestino horse growth since most have had a history of poor nutritional management. Together with their mothers, the foal stage has feeding based only on native forage and without food supplementation.

The studied animals are characteristic of the northeastern semi-arid landscape and well-suited to open-field breeding conditions. Our findings revealed that those animals born between 2019 and 2022 exhibited greater height and superior body weights. During this period, they experienced more regular rainfall, increasing forage availability in pastures.

In the present study, it was observed that adult females (full mouth) kept on native pasture were small (short stature) and had WH ranging from 123 to 126 cm, which was to the above statements about the importance of the availability of forages.

The relationship between height at withers and body length (Table 4) varies from 0.99 (females) to 1.01 (castrated and entirely males) and indicates that, in general, they are well-proportioned animals.

The average croup height of the females was equal to the height at the withers (Table 4), which is desirable for the animal's balance and, as established by ABCCN. Castrated and all males had WH greater than CH, 137 and 135 cm; 135 and 134 cm, respectively. Preferably, the horse should have equal CH and WH. When the height at the withers is higher than at the croup, the horse is called tall in front, and if the opposite is true, it is called short in front. Both cases constitute defects resulting from the abnormal opening of the anterior and posterior joint angles, more or less affecting the animal's gaits and resistance^12^.

### Morphometric indices

The DTI (*P* = 0.0347), CFI (*P* = 0.0066), COI (*P* = 0.0167), WCR (*P* = 0.0124), LOI2 (*P* = 0.0116), and WH/NL (*P* = 0.0082) showed a significant difference depending on sex (Table 5).

**Table 5:** Mean ± standard deviation and maximum and minimum values of the morphometric indices of the *Nordestino* horse in Paraiba state according to the animal´s sex.

Variables	Females (110)	Castrated males (121)	Entire males (37)	*P* value
Dactyl-thoracic index	0.115 ± 0.01b	0.117 ± 0.01ab	0.119 ± 0.01a	0.0347
Body index	88.26 ± 6.07	86.64 ± 5.90	87.65 ± 5.09	0.1107
Conformation index	2.30–1.24	1.82 ± 0.20a	1.74 ± 0.22b	0.0066
Compactness index	2.24 ± 0.39ª	2.25 ± 0.36a	2.08 ± 0.48b	0.0167
WCR	1.00 ± 0.02b	1.01 ± 0.02a	1.01 ± 0.01a	0.0124
Load index 1	99.44 ± 12.24	100.11 ± 14.79	96.46 ± 12.36	0.3693
Load index 2	168.71 ± 20.76ab	171.05 ± 19.82a	163.64 ± 20.96b	0.0116
Estimated body weight	300.99 ± 60.03ab	311.72 ± 57.31a	288.48 ± 65.01b	0.0912
Observed body weight	303.24 ± 57.88ab	309.05 ± 52.52a	281.78 ± 70.17b	0.0809
WH/TD	1.15 ± 0.15	1.14 ± 0.13	1.18 ± 0.14	0.4000
HL/NL	0.82 ± 0.06b	0.82 ± 0.06b	0.86 ± 1.04a	0.0082

Distinct letters in the row differ from each other by the *t*-test at a 5% significant level; WCR = relationship between height at withers and croup; WH/TD = ratio between withers height (WH) and thoracic depth (TD); HL/NL = ratio between head length/neck length.

The DTI presented a higher value for all males and was statistically similar to castrated males. The animals gave DTI above 0.115, classifying them as heavy traction horses. The females and castrated males showed higher CFI (1.78 and 1.82, respectively), below the ideal value for saddle horses, which is 2.1125. Even so, the animals are better suited to saddle than traction.

All animals in this study were classified as hypometric because they had a body weight (observed and estimated) (Table 5) below 350 kg, according to the classification of Torres & Jardim^9^, therefore suitable for services that require speed.

### Multivariate analysis of morphometric traits

Among the 11 morphometric measurements, only five were considered discriminating by the stepwise analysis. They were the OBW obtained by the tape, the height of the substernal void, cannon girth, body length, and withers height.

Observed body weight is a fundamental measurement of the animal's condition and general health. It is an essential indicator of body size and mass and can influence several morphological characteristics. Body length is a measurement that contributes to assessing the animal's overall size. It is relevant to understand the proportions and conformation of the horse, which can affect its suitability for different purposes, such as work or sport. Wither height is a measurement traditionally used to determine a horse's stature. Classifying the animal according to its breed and breed pattern is a vital characteristic.

Table 6 lists the animals' classifications in the different groups (sex) based on the morphometric variables evaluated. Among the 110 females considered, only 56.36% (62) were allocated to their group. The others were given to the group of castrated animals and all males. In other words, 43.36% of females have a phenotypic profile similar to males, which explains the high classification error of females. Castrated males differed from the different sexual categories since 79.33% of the animals in this group were correctly classified.

**Table 6:** Matrix classification of the animals into their groups (percentage of correct classifications on the principal diagonal).

Sex	Percentage	Female	Castrated male	Entire male
Female	56.36	62	38	10
Castrated male	79.33	23	96	2
Entire male	24.32	13	15	9

In the linear discriminant classification (Table 7), it is observed that the variable withers height presented greater weight, followed by thoracic perimeter and Cannon girth.

**Table 7:** Linear discriminant analysis of the variables selected by the stepwise method to classify animals into the three categories (females, castrated males, and entire males).

Variables	Female	Castrated male	Entire male
Head length	1.85	1.84	1.94
Head width	1.21	1.06	1.07
Neck length	− 0.17	− 0.19	− 0.24
Withers height	528.49	551.13	549.21
Croup height	44.70	32.10	36.53
Body length	6.28	− 2.53	− 2.56
Thoracic perimeter	82.18	79.69	82.49
Cannon girth	81.17	115.36	107.59
Substernal void height	− 0.51	− 0.44	− 0.57
Thoracic depth	0.97	0.99	0.95
Live weight	− 0.12	− 0.12	− 0.13
Constant	− 509.49	− 516.96	− 512.42

Thoracic perimeter and Cannon girth are necessary morphometric measurements in evaluating horses, as they provide valuable information about the animal's physical conformation and ability to perform different activities.

Almost the majority of the remaining were classified in the female group, and only two animals were classified as entire males (Table 7). The highest proportion of animals classified incorrectly, that is, in a group different from their group of origin, were castrated males (75.68%), followed by females (43.64%) and entire males (24.67%).

## Discussion

### Characterization of the hearing system

Unfortunately, many of the interviewees reported that animals return with physical and psychological problems. According to Hering^13^, in the traditional taming model, the peculiarities of the animal's natural behavior are not considered, and little care is given to mental and, in some cases, physical health. In this process, the horse is often driven to exhaustion instead of establishing a trusting connection with the animal.

### Phenotypic profile of animals

In terms of proportionality, when dividing the height at the withers of all classes of animals by 2.5, as described by Camargo & Chieffi^14^, the animals had a harmonic head, that is, a shorter head, which is desirable, according to the ABCCN^11^ breed standard. The width of the head must be equivalent to a third of its length, according to the Eclectic System of Linear Proportions by Lesbre, cited by Torres & Jardim^9^, demonstrating that the animals presented this proportionality, especially entire males.

In the substernal void height, castrated males presented a higher value, which allowed classifying all animals as “long-legged,” with an elevation at the substernal void greater than that of the thoracic depth. The males had bodies further from the ground than the others and were more suitable for speed tests^12^.

According to ABCCN^11^, the ideal WH for females is 135 cm, with a minimum of 127 cm and a maximum of 143 cm. The differences observed in many studies for the same breed are due to the other climate conditions and food availability in the Caatinga ecosystem.

Melo et al.^1^ verified adult males from the Nordestino horse in Pernambuco with an average WH of 132.31 cm, ranging from 122 to 147 cm. Travassos^15^ verified WH for males over three years at 136 cm and females older than three years at 131 cm. Dias^16^, on the other hand, observed an average WH for males of 128.60 cm, females of 125.86 cm, and castrated males of 127.64 cm. For females, the average WH was 135 cm, varying from 123 to 143 cm.

The relationship between withers, height, and body length must be equal^14^. Melo et al.^1^ observed that this relationship was 0.97 for females and 1.01 for males.

Both males and females are classified as small animals, which, according to Torres & Jardim^9^, must have a withers height below 1.50 m. The differences found for the same breed by different authors must be related to the sampling effect and ecological conditions. They are samples of different sizes obtained from animals of varying ages under other management systems and collected by several evaluators in different locations^1^.

At birth, the foal already presents appreciable linear growth with around 60–70% of the withers height of an adult animal, reaching 88% at 12 months, 95% of its maximum growth at 24 months, and 100% at 60 months, in average, in its WH^17^.

In the prenatal phase, the development of the skeleton and organs occurs. After birth, there is an acceleration of growth and an increase in tissue deposition until puberty. From puberty onwards, around two and a half to 3 years of age, muscle deposition ceases, and fat deposition occurs; there is a slowdown in growth, and the animals reach mature weight and size^18^.

Many factors can interfere with animals' pre- and post-natal growth, such as nutrition and feeding, stressful environmental conditions, mother's age, breed, sex, climate, birth year, geographic location, and training. Among these, nutrition plays the most crucial role in the success of all stages of animal life, as it influences placental development and, consequently, fetal and postnatal growth, particularly maternal nutrition^5^. Deficient management conditions lead to growth retardation, and the nutritional impact on the foal's development manifests itself in several ways, from slowed global growth to changes in metabolic and structural characteristics usually displayed at an older age^19^. Inadequate feeding of the Nordestino horse can contribute to an unfair comparison of this breed against others^20^. Animals have a genetically predetermined growth pattern, but the actual growth of animals involves, in addition to genetic factors, environmental factors. In this way, animals grow to a maximum size according to their breed^21^.

Travassos^15^ recorded an average croup height of 136.20 cm in male animals and 131.70 cm in females in Pernambuco. On the other hand, Dias^16^ observed smaller values in animals from Piauí, noting 128.42 cm for females, 127.48 cm for castrated males, and 127.65 cm for entire males, all of which were smaller than the measurements observed in our study.

The cannon girth in this study was more significant in males than in females. It allowed the characterization of males (entire or castrated) as having thicker shins, indicating a more robust and sturdy build, providing a solid foundation crucial for their activity performance. These results corroborate with Melo et al.^1^, who found sexual dimorphism for cannon girth in Nordestino horses in Pernambuco state, with the cannon girth value of males (castrated or entire) being more significant than that of females.

### Morphometric indices

Our results differ from those observed by Travassos^15^, which were lower for the DTI, 0.110 and 0.113 for females and males, respectively. Melo et al.^1^ observed DTI in animals from Piauí state of 0.115 and 0.116 for females and males (castrated and entire), respectively.

The characterization of most animals as being suitable for heavy traction does not reflect the biotype of the Nordestino horse, which is light in build and weighs, on average, less than 350 kg. Thus, although the indices indicate the animal's fitness, they should not be unique evaluation and characterization parameters for that genotype.

Based on the BI value, the animals were classified as mediolines^4^ and, according to Torres & Jardim^9^, with an intermediate aptitude for activities that require speed and strength. Medioline horses have balanced body proportions, are ideal for riding, and can be used for saddle activities^22^. Medioline horses were also observed by Dias^16^ and Melo et al.^1^, who studied animals from Pernambuco and Piauí states.

The Eclectic System of Linear Proportions, proposed by Lesbre^23^ and cited by Souza et al.^24^, has been used for several decades to study the proportions of saddle horses.

This study is based on the relationships between the different regions of the body and the assemblage formed by them. This relationship is presented as follows: the WH and croup, and the length of the body are equivalent to two and a half times the length of the head, and the length of the neck and shoulders have the same value as the length of the head. The animals in this research are recommended for saddle riding using the eclectic system.

Melo et al.^1^ found average values of 1.7081 and 1.7558 for the conformation index, for males and females, respectively, of the Nordestino horse in Juazeiro, in Bahia. Serra^25^, evaluating animals from the Baixadeiro horse, found a value of 1.65, close to those obtained in this study for animals from the state of Piauí, and characterized the Baixadeiro horse as suitable for saddle riding.

The compactness index presented a higher value for females and castrated males. However, the value was below what Torres & Jardim^9^ determined to classify as a saddle animal, below 2.60. The values found in this research were similar to those of Melo et al.^1^.

With LOI1, it was observed that the animals have greater load capacity on their backs without excessive effort when working at a trot or gallop, with an overall average support load of 98.67 kg. According to LOI2, the support load on the back is 167.80 kg, which allows the animals to work at a pace without excessive effort. A similar value was found by Melo et al.^1^ in animals from the state of Pernambuco (168.26 kg).

The differences found for the same breed by different authors must be associated with the sampling effect and ecological conditions. They are samples of various sizes, obtained from animals of different ages, under other management systems, and collected by different measurers in different locations.

However, it is emphasized that the hypermetric size of Nordestino horses seems to be advantageous due to their speed, agility, and resistance during cattle management activities in pastures, mainly in the caatinga, and, perhaps, it is one of the preferred sizes by breeders and cowboys for work or to practice “Pega de Boi no Mato”– Cattle Capture in the Brush, in free translation—a sporting activity traditionally practiced in the Northeast region.

On the other hand, there is an expectation on the part of breeders and technicians, demonstrated during this study, for the emergence of horses of this breed with larger sizes and specific gaits (walk, trot, gallop) or ambling (walk, rack, or tolt) so that they can participate in other equestrian activities, such as “*free de ouro*,” “*vaquejadas*” [Bull-catching] and hiding tours.

However, the emergence of animals with some of these characteristics will be, at least, in the medium term, as animals of this breed have not received the attention they deserve for more than 200 years. Therefore, there is now an urgent need for a strong effort on the part of the actors involved (breeders, breeders' association, technicians, researchers, development, teaching, and S&T institutions, among others) to develop actions aimed at improving the conditions of management (nutritional, reproductive and sanitary) that result in herds with animals subject to animal selection and genetic improvement.

## Conclusions

The remaining Nordestino horses have morphological characteristics within the breed standard. One problem identified is that the larger males are being castrated, leaving the smaller ones as entire males.

The predominant breeding system in the region is extensive, with animals consuming native forage and deficient breeding management.

It was observed that a high percentage of the females and all males were classified into different groups because the animals had very similar phenotypes in terms of withers height. The variables that best discriminate animals are live weight, substernal void height, Cannon girth, body length, and withers height.

Most of the animals evaluated met the standards established for the breed. Larger males are often being castrated, while smaller ones remain as whole males. The extensive breeding system is the predominant one. The variables that best discriminate animals are live weight, substernal void height, Cannon girth, body length, and withers height.

Identifying discriminant variables helps us understand the morphology of the Northeast horse, provides practical tools for future studies with the breed, and provides tangible parameters for the careful selection of sires.

Practical interventions and sustainable management strategies can be developed based on the results of this research. This includes guidance for breeders on reproductive practices to conserve the breed's genetic diversity and distinctive morphological characteristics.

This research contributes to understanding the morphological characteristics of the Nordestino horse breed and highlights the importance of responsible and sustainable management practices to ensure the permanence of the breed.

## Data Availability

The datasets analyzed during the current study are available from the corresponding author on reasonable request.
